# Acute Phlegmonous Gastritis: Insights Into Etiology, Diagnosis, and Clinical Management

**DOI:** 10.7759/cureus.100916

**Published:** 2026-01-06

**Authors:** Ian Seng Lam, Hoi Ip Leong, Tam Fei Chang, Choi Chu Lei

**Affiliations:** 1 Emergency Department, Conde S. Januário Hospital, Macao, CHN

**Keywords:** acute abdomen, acute phlegmonous gastritis, diabetes, metabolic acidosis, septic shock

## Abstract

Acute phlegmonous gastritis is a rare and life-threatening form of gastritis characterized by suppurative inflammation of the gastric wall. We present the case of a 66-year-old female with a history of diabetes and hypertension who presented with abdominal pain, vomiting, and diarrhea, progressing to refractory shock and severe metabolic acidosis. Abdominal imaging revealed diffuse thickening of the gastric wall with surrounding fat strands, suggesting acute phlegmonous gastritis. Despite aggressive resuscitation and critical care management, the patient's condition continued to deteriorate, and she ultimately succumbed to her illness. The importance of this case is early recognition, giving fluid resuscitation and broad empirical antibiotics, and prompt surgical intervention in acute phlegmonous gastritis, a condition with a high mortality rate.

## Introduction

Acute phlegmonous gastritis (APG) is an uncommon yet devastating suppurative infection of the stomach's submucosa and muscular layers. Its rarity often leads to delayed diagnosis, contributing to its high mortality rate [1]. Historically, mortality rates were as high as 92% in the pre-antibiotic era. While antibiotic therapy has reduced mortality to approximately 48%, it remains substantial, underscoring the severity of this condition [1].

APG primarily affects the population aged 45-74 years old, with male predominance. The underlying etiology of APG is unclear; an immunocompromised condition associated with diabetes mellitus, malignancy, chemotherapy-related neutropenia, acquired immunodeficiency syndrome (AIDS), alcoholism, and immunosuppressive drugs are considered important risk factors [2].

The clinical presentation of APG is often non-specific, mimicking common abdominal diseases, which can lead to delays in initiating appropriate treatment. Diagnosis primarily relies on a high index of suspicion supported by characteristic findings on computed tomography (CT) scans, such as diffusely thickened gastric walls and perigastric fat stranding. Early broad-spectrum antibiotic therapy can be curative in some cases. However, even with the correct diagnosis and antimicrobial therapy, the mortality rate remains high at 27-40% [2]. Most of the treatment for severe cases involves a combination of broad-spectrum antibiotics with urgent surgical intervention.

This report describes a fatal case of acute phlegmonous gastritis (APG) in a 66-year-old female, highlighting the necessity for early detection and decisive management. The patient presented with septic shock, supported by diagnostic findings of marked leukocytosis on laboratory analysis and diffused gastric wall thickening on computed tomography. This case reinforces that prompt multidisciplinary evaluation, including gastroenterology for possible urgent endoscopy and surgical consultation, is critical. Early and expedient surgical intervention remains the cornerstone of effective treatment and is essential to improve the poor prognosis associated with this rapidly progressive condition.

## Case presentation

A 66-year-old female with a history of diabetes and hypertension presented to the emergency department with a chief complaint of abdominal pain, vomiting, and diarrhea for half a day. The patient's family reported that she had failed to check her blood pressure at home for the past hour. Upon arrival, the patient was drowsy; vital signs showed the patient's temperature was 36.2°C, blood pressure was 74/36 mmHg, and pulse was 120 times/min. Physical examination found tenderness in the epigastric region, no muscle guarding, and weak bowel sounds.

Initial laboratory findings revealed severe metabolic acidosis, leukocytosis, acute kidney injury, and hypernatremia (Table 1). Based on the arterial blood gas analysis and subsequent blood test results, along with the clinical presentation, the patient was considered to have septic shock, intra-abdominal infection, hypovolemia, hypernatremia, and acute kidney injury.

**Table 1:** Laboratory result at emergency department admission pCO₂: Partial Pressure of Carbon Dioxide, pO₂: Partial Pressure of Oxygen, RBC: Red Blood Cell, MCV: Mean Corpuscular Volume, MCH: Mean Corpuscular Hemoglobin, MCHC: Mean Corpuscular Hemoglobin Concentration, RDW: Red Cell Distribution Width, WBC: White Blood Cell

Test Name	Result	Reference Range
Arterial Blood Gas		
pH	7.06	7.35 - 7.45
pCO₂	16 mmHg	35 - 45 mmHg
pO₂	145 mmHg	80 - 100 mmHg
Base Excess (BE)	-28 mmol/L	-2 to +2 mmol/L
Lactate (Lac)	11.4 mmol/L	< 2.0 mmol/L
Complete Blood Count		
RBC	6.4 x10¹²/L	4.5 - 5.9 x10¹²/L
Hemoglobin (Hb)	17.6 g/dL	13.5 - 17.5 g/dL
Hematocrit (Hct)	58.3 %	40 - 52 %
MCV	91.1 fL	80 - 100 fL
MCH	27.5 pg	27 - 33 pg
MCHC	30.2 g/dL	32 - 36 g/dL
RDW	15.7 %	11.5 - 14.5 %
WBC	38.8 x10⁹/L	4.0 - 10.0 x10⁹/L
Neutrophils	31.4 x10⁹/L	2.0 - 7.5 x10⁹/L
Eosinophils	0.2 x10⁹/L	0.0 - 0.5 x10⁹/L
Basophils	0.2 x10⁹/L	0.0 - 0.1 x10⁹/L
Lymphocytes	5.5 x10⁹/L	1.0 - 4.0 x10⁹/L
Monocytes	1.5 x10⁹/L	0.2 - 0.8 x10⁹/L
Platelets	586 x10⁹/L	150 - 450 x10⁹/L
Chemistry & Inflammation		
C-Reactive Protein (CRP)	0.14 mg/dL	NEG <=0.5 mg/dL
Procalcitonin (PCT)	0.16 ng/mL	< 0.06 ng/mL
Urea	15.2 mmol/L	2.9 - 8.2 mmol/L
Creatinine	113 µmol/L	45 - 84 µmol/L
Uric Acid	233 µmol/L	143 - 339 µmol/L
Glucose (Random)	20.48 mmol/L	< 11.1 mmol/L (random)
Sodium	152 mmol/L	136 - 145 mmol/L
Potassium	3.2 mmol/L	3.5 - 5.1 mmol/L
Chloride	116 mmol/L	98 - 107 mmol/L
Calcium	1.35 mmol/L	2.20 - 2.55 mmol/L
Phosphorus	2.04 mmol/L	0.81 - 1.45 mmol/L
Magnesium	0.71 mmol/L	0.66 - 0.99 mmol/L
Total Protein	24 g/L	64 - 83 g/L
Albumin	7 g/L	35 - 52 g/L
Liver & Pancreatic Enzymes		
Total Bilirubin	1 µmol/L	< 15 µmol/L
Aspartate Aminotransferase (AST)	38 U/L	<= 32 U/L
Alanine Aminotransferase (ALT)	17 U/L	<= 33 U/L
Creatine Kinase (CK)	171 U/L	< 170 U/L
Lactate Dehydrogenase (LDH)	159 U/L	135 - 214 U/L
Amylase (Total)	71 U/L	28 - 100 U/L
Lipase	76 U/L	13 - 60 U/L

Notably, the patient presented with marked leukocytosis and neutrocytosis in the absence of elevated C-reactive protein (CRP) and procalcitonin (PCT). This discordance may be explained by the different kinetics of these biomarkers, where the rapid and fulminant onset of sepsis outpaced the typical lag time for CRP and PCT elevation. The concomitant presence of hypoalbuminemia further supports the diagnosis of a severe, systemic inflammatory response, often resulting from capillary leakage and hepatic reprioritization of protein synthesis. Severe metabolic acidosis with hyperlactatemia corroborates this timeline of acute clinical deterioration.

The patient was immediately resuscitated with intravenous fluids, empirical antibiotics, and a central venous line was inserted. She was intubated and placed on mechanical ventilation due to her progressive decrease in consciousness. After that, she was admitted to the ICU. Despite fluid resuscitation and the initiation of vasopressor support with norepinephrine, the patient's hemodynamic status remained unstable.

**Figure 1:**
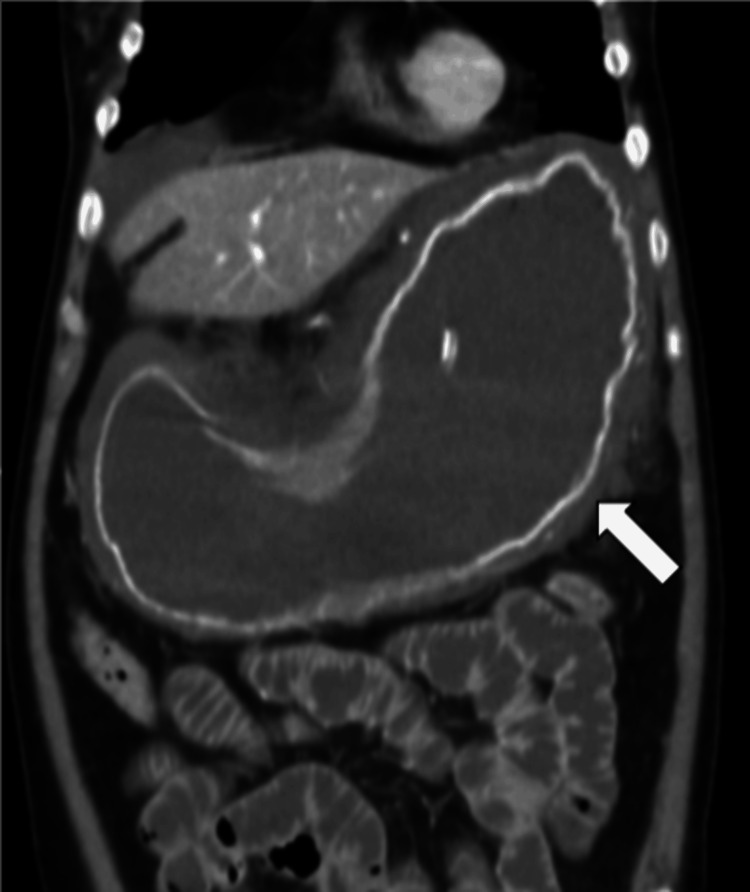
Diffuse thickening of the gastric wall with surrounding fat stranding detailing in the abdomen contrast-enhanced CT scan coronal view (white arrow).

**Figure 2:**
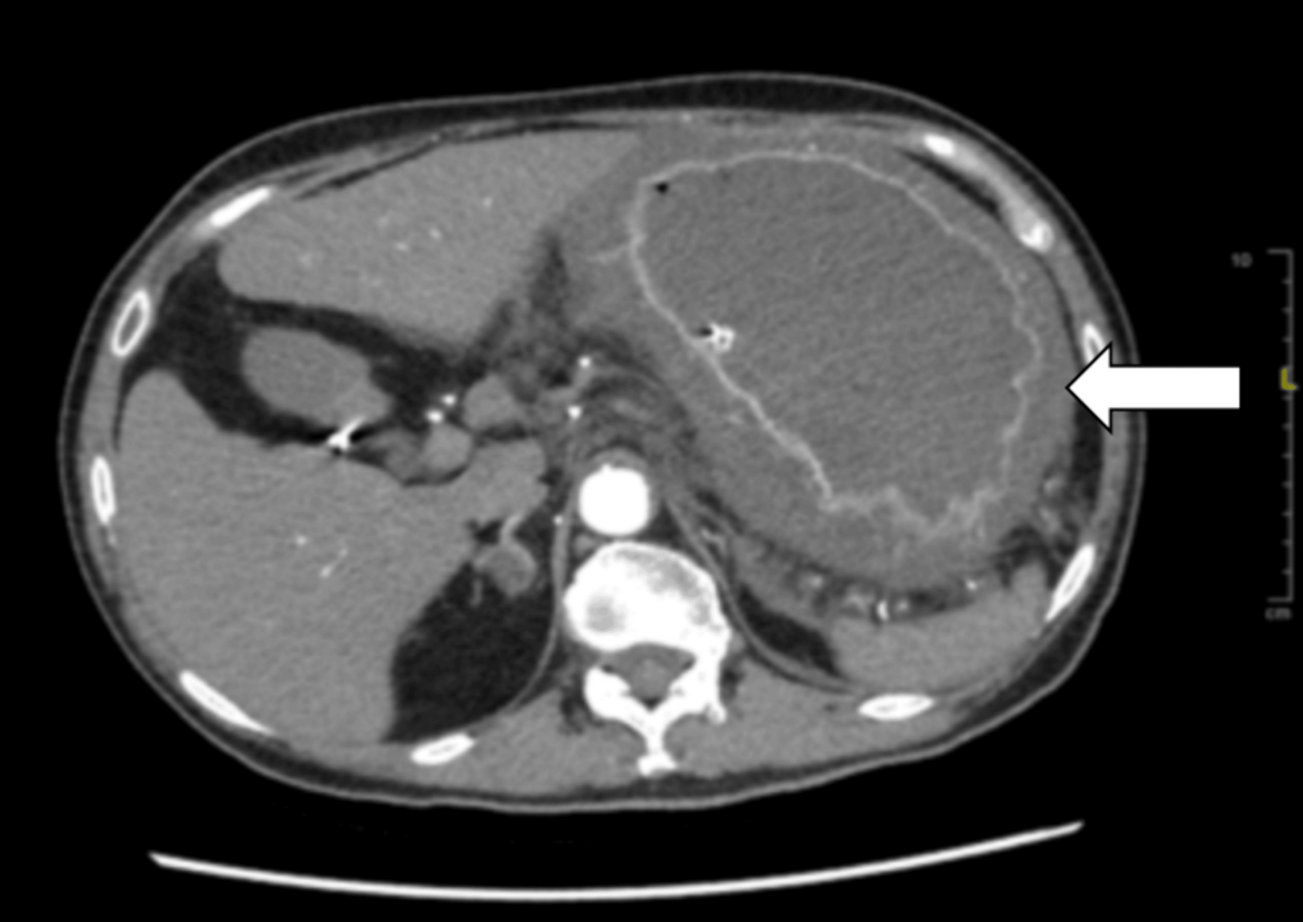
Diffuse thickening of the gastric wall with surrounding fat stranding detailing in the abdomen contrast-enhanced CT scan axial view (white arrow).

Abdominal and pelvic computed tomography (CT) revealed diffuse gastric wall thickening with surrounding fat stranding, suggestive of acute phlegmonous gastritis (Figures 1, 2). No evidence of pneumoperitoneum or significant ascites was present. The differential diagnosis for diffuse gastric wall thickening on CT includes both benign and malignant processes. Malignant etiologies such as gastric adenocarcinoma or lymphoma typically present with more focal or nodular thickening and are often associated with lymphadenopathy. Benign inflammatory conditions, like severe erosive gastritis or Crohn's disease, can have similar appearances but usually lack the pronounced perigastric inflammatory fat stranding seen in this case. Other considerations, such as gastric ischemia or portal hypertensive gastropathy, were deemed less likely given the clinical context and the absence of supporting vascular findings. The combination of diffuse thickening, intense perigastric fat stranding, and the absence of a focal mass or significant lymphadenopathy is highly characteristic of acute phlegmonous gastritis, supporting the primary radiological diagnosis.

Following urgent evaluation with specialists in gastroenterology and surgery and subsequent review of the CT images in conjunction with the patient's clinical presentation, the consensus diagnosis was acute phlegmonous gastritis. Urgent endoscopy was deferred due to the patient's profound hemodynamic instability, with management priorities focused on aggressive fluid resuscitation, empirical broad-spectrum antibiotic therapy, and preparation for urgent surgical exploration.

However, the patient's hemodynamic status continued to deteriorate, and the surgical intervention was ultimately canceled due to the refractory nature of her shock. Despite maximal medical support, including the use of additional vasopressors and sodium bicarbonate for the severe metabolic acidosis, the patient's condition continued to worsen, and she was ultimately certified dead.

## Discussion

This case report details a 66-year-old female patient with acute phlegmonous gastritis (APG), a rare and rapidly progressive bacterial infection of the gastric wall. The patient presented with an acute abdomen and systemic symptoms that swiftly evolved into septic shock. Key findings included characteristic diffuse gastric wall thickening with perigastric fat stranding on abdominal CT imaging and notable laboratory findings of marked leukocytosis alongside severe metabolic acidosis and acute kidney injury. Despite immediate aggressive resuscitation, broad-spectrum antibiotics, and intensive care support, the patient developed refractory shock and succumbed to the illness. This fatal trajectory underscores the diagnostic challenges and critically poor prognosis associated with APG, setting the stage for a discussion on its pathophysiology and management dilemmas.

Management commenced with fasting, rehydration, and empirical antibiotic therapy. However, the patient's condition rapidly declined, requiring urgent assessments by both the gastroenterology and general surgery teams, who recommended immediate surgical intervention. Unfortunately, the patient's hemodynamic status worsened, necessitating the cancellation of the surgical procedure due to refractory shock. Despite maximal medical support, including the administration of additional vasopressors and sodium bicarbonate to counteract severe metabolic acidosis, her clinical condition continued to deteriorate. Intensive efforts to stabilize her were ultimately unsuccessful, and the patient was certified dead.

APG was first described by Cruveilhier in the early 19th century, with an average of only one reported case per year over the past six decades. This condition can affect individuals of all ages but is most commonly seen in adults aged 50 to 70 years, with a male-to-female ratio of 2:1. Early diagnosis and surgical intervention have been shown to improve survival in some cases [2].

Despite the widespread use of antibiotics, APG remains a rare but highly emergent and rapidly progressing condition. Due to its high mortality rate, early recognition and prompt intervention are crucial. Additionally, antibiotic therapy alone can be effective if initiated early [3].

The exact etiology of APG is not fully understood, but it is believed to be caused by the invasion of the gastric wall by pathogenic bacteria. Approximately 70% of cases have been associated with hemolytic *streptococcus*, followed by *Staphylococcus aureus*, *Streptococcus pneumoniae*, and *Enterococcus*. Bacterial invasion of the gastric wall can occur through various mechanisms, such as gastric ulcers, chronic gastritis, direct invasion from the pharynx, hematogenous spread from respiratory or other infections, or lymphatic spread from cholecystitis or peritonitis. The risk factors for APG are old age, DM, immunocompromise, peptic ulcers, septicemia, and instrumental use such as OGD [4]. Microbiological culture of gastric tissue biopsies is instrumental in identifying the causative organism. This diagnostic step, combined with pathological examination, is crucial for distinguishing APG from mimicking conditions, including gastric carcinoma, MALT lymphoma, gastrointestinal stromal tumor (GIST), leiomyoma, and carcinoid tumors [5].

Radiological and endoscopic evaluations are vital for a rapid presumptive diagnosis. Characteristic findings on abdominal ultrasonography or CT include marked gastric wall thickening and infiltration of the surrounding fat. Endoscopic findings may include fibrinopurulent exudates lining the stomach, loss of rugae, edematous mucosa, and superficial ulcerations [6]. These findings enable a strong working diagnosis, thus facilitating the immediate commencement of therapy to enhance patient survival prospects. 

The clinical presentation of APG is often nonspecific, with abdominal pain, nausea, vomiting, and fever being the most common symptoms. However, as seen in our case, the condition can rapidly progress to septic shock and severe metabolic acidosis, leading to multiorgan dysfunction and a high risk of mortality. Prompt recognition and timely surgical intervention are crucial in the management of APG. The primary treatment involves aggressive resuscitation, broad-spectrum antibiotic therapy, and surgical debridement or gastrectomy to remove the infected gastric tissue. In some case reports, gastric lavage with nasojejunal feeding is also an alternative management of APG [7]. Complications such as delayed gastric perforation have been reported [8]. In cases where the patient's condition is too unstable for immediate surgery, temporary stabilization with antibiotics and supportive care may be attempted, but the prognosis is often poor.

In the present case, the patient's refractory shock and severe metabolic acidosis precluded definitive surgical management, and she ultimately succumbed to her illness despite maximal medical support.

The importance of this case is early recognition. If the patient presents with non-specific acute gastroenteritis symptoms such as vomiting and diarrhea, blood gas shows unexplained progressive metabolic acidosis, or they present symptoms of sepsis or hemodynamic instability, early abdomen-pelvic CT or gastric endoscopy, empirical antibiotic treatment, and prompt surgical intervention are necessary in acute phlegmonous gastritis, as delayed treatment is associated with a high mortality rate. The primary treatment involves aggressive resuscitation, broad-spectrum antibiotic therapy, and surgical debridement or gastrectomy to remove the infected gastric tissue [9].

## Conclusions

Acute phlegmonous gastritis (APG) remains a rare but highly fatal surgical emergency. This case, consistent with the broader literature, underscores the critical association between diagnostic challenges, rapid clinical deterioration, and poor outcomes. Our experience, along with prior reports, suggests that outcomes are likely influenced by the timing of recognition and intervention. A high index of suspicion is crucial when evaluating septic patients with significant, unexplained metabolic acidosis and abdominal symptoms. Prompt confirmation with emergent radiology imaging or endoscopy, followed by aggressive resuscitation and empiric broad-spectrum antibiotics, is essential to stabilize the patient as a bridge to potential definitive therapy. Early surgical consultation is paramount, as existing evidence indicates that operative intervention (gastrectomy or debridement) is often required for source control and may be curative when hemodynamically feasible. Ultimately, improving outcomes for this devastating condition may hinge on its early consideration and a coordinated, multidisciplinary approach aimed at intervention before the onset of irreversible shock.
